# A new affinity matrix weighted k-nearest neighbors graph to improve spectral clustering accuracy

**DOI:** 10.7717/peerj-cs.692

**Published:** 2021-09-06

**Authors:** Muhammad Jamal Ahmed, Faisal Saeed, Anand Paul, Sadeeq Jan, Hyuncheol Seo

**Affiliations:** 1 The School of Computer Science and Engineering, Kyungpook National University, Daegu, Daegu, South Korea; 2 Department of Computer Science & IT, University of Engineering Technology Peshawar, Peshawar, Peshawar, Pakistan; 3 School of Architectural, Civil, Environmental and Energy Engineering, Kyungpook National University, Daegu, Daegu, South Korea

**Keywords:** K-nearest neighbors, Spectral clustering, Eigen decomposition, Affinity matrix

## Abstract

Researchers have thought about clustering approaches that incorporate traditional clustering methods and deep learning techniques. These approaches normally boost the performance of clustering. Getting knowledge from large data-sets is quite an interesting task. In this case, we use some dimensionality reduction and clustering techniques. Spectral clustering is gaining popularity recently because of its performance. Lately, numerous techniques have been introduced to boost spectral clustering performance. One of the most significant part of these techniques is to construct a similarity graph. We introduced weighted k-nearest neighbors technique for the construction of similarity graph. Using this new metric for the construction of affinity matrix, we achieved good results as we tested it both on real and artificial data-sets.

## Introduction

Clustering data is a prevailing technique used in unsupervised learning; its goal is to breakdown data into clusters (Ünal, Almalaq & Ekici, 2021) in a way that representatives of the identical cluster are better identical to each other, conferring to some resemblance measure (Frate et al., 2021) than any two members from two different groups. The categorical partition of recent clustering techniques can be as follows: hierarchical clustering, partitioning clustering, grid-based clustering, and density-based clustering, correspondingly. Although the preceding clustering techniques displayed decent achievement, but those methods in its applicability to big data because of their highly computation complexity are limited (Xu & Zhang, 2004). In real-world complications diverse applications of clustering are revised *e.g*. in Danesh, Dorrigiv & Yaghmaee (2020). The procedure is effectively cast-off *e.g*. in management for hazard appraisal Bharti & Jindal (2020) and Jain (2010) or in portfolio management (Sanchez-Silva, 2009). Even though several clustering approaches have been suggested in the late periods, see *e.g*. Chen et al. (2003) or Zhang & Maringer (2010), a prevalent clustering approach which is worthy of dealing with any kind of clustering problem; subsequently, the real-life clusters may be of diverse densities, random complicated shapes and instable sizes. Path-based clustering and spectral clustering are dualistic developed clustering methods recently and these both have conveyed remarkable outcomes in a numeral of stimulating clustering problems, although path-based and spectral clustering both are not sufficiently robust counters to outliers and noise in the data. Regardless of the auspicious achievement of path-based and spectral clustering algorithms testified on some problematic data sets, there are some certain situations when both of these algorithms do not achieve that auspicious performance. Choosing affinity matrix is of great significance, it will be the reason whether the clustering results will be good or bad. Affinity matrix is generally defined in a similar manner to the Gaussian kernel based on inter-point Euclidean distance in the input space. Clustering data is an essential and complex problem in pattern recognition, computer vision and data mining, such as gene analysis, object classification, image segmentation and study of social networks. The intention of clustering data, also called cluster analytics or analysis, is to observe the ordinary grouping(s) of a collection of objects, points, or patterns (Jain, Murty & Flynn, 1999). Webster expresses the analysis of cluster as “a classification method using statistics for determining whether the entities of a population plunge into unlike clusters by formulating quantitative assessments of numerous features” (Xu & WunschII, 2005). There is a wide use of cluster analysis in abundant applications, inclusive of image processing, data analysis, market research, and pattern recognition (Jain, 2005; Larose, 2005). Dynamic development is going on in data clustering, Statistics, Data Mining, Biology, Spatial Database Technology, Marketing and Machine Learning are the main contributing zones of research. Cluster analysis has developed as an extremely vigorous topic in data mining research in the recent times and the reason to this is the enormous volume of data collected in databases (https://www.merriam-webster.com/). Coming to clustering techniques developed in recent time, one of the utmost extensively used clustering is spectral clustering; the reason behind its extensive usage includes solid mathematical ground-works and minimal norms on data distribution. Spectral clustering, in its unique unsupervised form, utilizes pairwise similarity to cluster samples, which is further expressed and concluded through a graph Laplacian matrix, although in this system there is no means to certify that the consequential groups or clusters resemble to the other user-defined designs or semantic of categories and cadres in the data. The spectral clustering technique is smooth and straightforward to implement, while in performance it dominates conventional clustering techniques like the k-means clustering algorithm. Principally, there are three main steps in spectral clustering: pre-processing, decomposition, and grouping. A similarity graph and its adjacency matrix are forged for the data set in the first step (pre-processing) of spectral clustering. In the second step(decomposition), during the eigen vectors of the matrix presentation of data set is changed. In the third step (grouping), groups or clusters are mined from the fresh depiction.

The rest of our manuscript is organised as follows: the next section concisely briefs the existing work of constructing similarity graph and spectral clustering algorithms. In “Proposed Approach”, we discussed the proposed approach. After that we have the “Results and Discussion” section, and followed by the concluded remarks.

## Related Work

**Figure 1:**
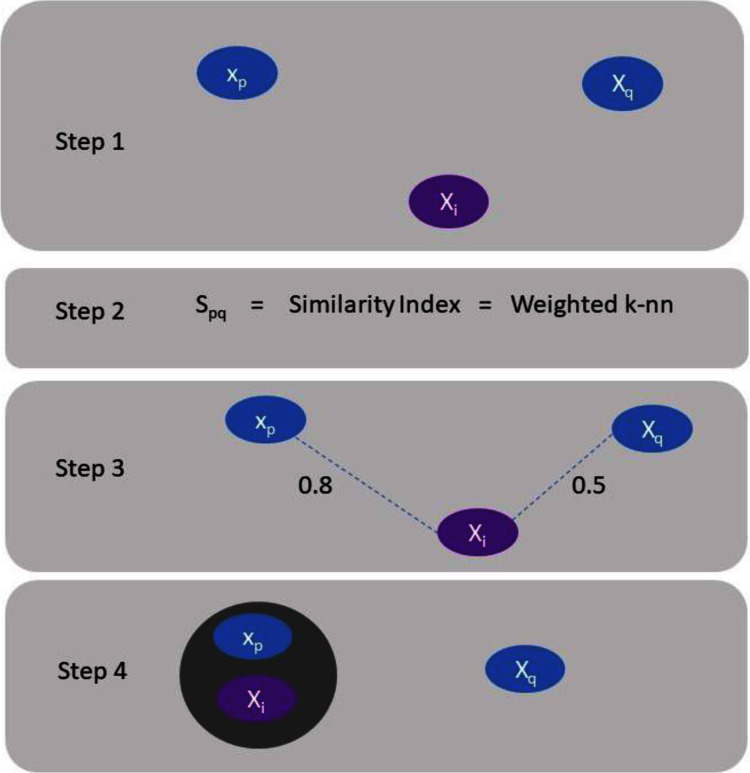
Explanation of assigning a data point to a cluster.

Clustering of data have been considered for an extensive period of time, and a complete overview to the construction of graphs techniques can be found in Sumathi & Esakkirajan (2001). The following section comprises of brief review and numerous current approaches for spectral clustering, graph partitioning and construction (Han & Kamber, 2000). Spectral clustering literature can be categorized into two classes (Sumathi & Esakkirajan, 2001). One is the emphasis on clustering data in the presence of a similarity graph when it is given, and the second one is the emphasis on the construction of the similarity graph using a precise spectral clustering technique. Consider we have Z = (*x*_1_, . . .*x*_*n*_) a set of data points and some concept of similarity *s*_*pq*_ ≥ 0 among all subsets of data points *x*_*p*_ and *x*_*q*_. The clustering instinctive objective is to categorize the data points into numerous clusters in such a way that points in the identical cluster are related and points in unlike clusters are different to individual ones (Chen, Fang & Saad, 2009). If the problem is not to have additional material (information) than similarities (Rathore et al., 2018) among data points, an appropriate technique of characterizing the data is to construct a similarity graph G = (V, E). In the graph every vertex “*v*_*i*_” signifies a data point “*x*_*p*_”. A certain threshold is set to check the connectivity between two vertices. If the similarity between two corresponding data points “*x*_*p*_” and “*x*_*q*_” is positive or larger than the certain threshold then edge is weighted by *s*_*pq*_ accordingly. This situation is illustrated by Fig. 1.

Now we can reformulate the complication of clustering by means of similarity graph. So, the utmost desire or need is to find segregated region (partition) (Saeed, 2018) of the graph to such a degree that edges among diverse groups have minor weights. What this means is that points in different groups are different from each other. While the edges inside a cluster have larger weights, this acknowledges that points inside the identical cluster are similar to each data point. In the primary class, there are various research that advances the performance and efficiency of clustering Macleod, Luk & Titterington (1987). In particular, Gul, Paul & Ahmad (2019), Saeed (2019) and Belkin & Niyogi (2004) find the amount of principal Eigen vectors and segregate the data using latent tree models. Bentley (1975) Used NMF (non-negative matrix factorization) with spectral clustering, and recommended non negative and sparse spectral clustering technique. Bentley (1980) proposed Eigen-vector selection algorithm with novelty in informative/relevant, which defines the total numbers of clusters. Bentley (1980) introduced a technique with the idea of divide-and-conquer style for assembling approximate the k-nearest neighbour graph. In this technique, the data-points are recursively divided into subgroups and are overlapped, then a single k-nearest neighbour graph is constructed on every individual small subgroup. The concluding graph is fabricated by integrating all those subgroup graphs organised overall by means of overlapping fragments. Analytically, it was reported by the authors that their approach had O(*dn*^1.22^) time complexity. Lately, Bentley (1980) introduced an alternate effective method by means of the same kind of idea. But the main difference is that the datasets are without overlapping recursively divided. To escalate the k-nearest neighbour reminiscence, it assembles several elementary graphs by reiterating the division technique for numerous times period. To form a decent division, the two approaches use convention direction to segregate the data set. The k-nearest neighbour search is an instance-based learning problem, which states in the training phase we cannot promote query points. Nonetheless, construction of k-nearest neighbour graph is an exude problem which has all the query points at his hand. Therefore, construction of such a graph is uncomplicated in routine, and we could take benefits of its various characteristic to propose more effective algorithm.

## Proposed Approach

In this section we introduce a new similarity graph technique for spectral clustering. We propose a weighted k-nearest neighbors approach for spectral clustering. The reason behind using weighted k-nearest neighbors approach is the use of hyper-parameter k, which influences the performance of k-nearest neighbors. Spectral approach has two significant roles that makes it very attractive. First, it provides us with a mathematically-sound formulation (Charikar, 2002). The second advantage is computation speed. Spectral methods have become standard techniques in algebraic graph theory (Wang et al., 2012). The most widely used techniques utilize eigenvalues and eigenvectors of the adjacency matrix of the graph. Further, nowadays the significance has deviated slightly to the spectrum of the intently associated Laplacian. Indeed, Mohar (Chung, 1997) wrote that the Laplacian spectrum is more essential than that of the adjacency matrix associated field where the spectral technique has been promoted comprising ordering (Chung, 1997), partitioning (Mohar, 2004) and clustering (Juvan & Mohar, 1992). Moreover, the areas like clustering, partitioning, and ordering, unlike graph drawing practice discrete quantization’s of the eigenvectors, which operates the eigenvectors deprived of any amendments.

### Techniques used for clustering

There are two extensive techniques for clustering:

- Compactness
- Connectivity
  1. Data points that are positioned nearby to one another are clustered in the same group and are compact everywhere the centre of cluster. Through distance between the observations, the proximity can be measured, like k-Means.
  2. Connected data points or immediately next to each other are clustered together. Even though the remoteness between two data points is minor, if there is no connection, the data points are not clustered into a group. Spectral clustering is kind of method that pursue this technique.

### Weighted-KNN based spectral clustering

**Figure 2:**
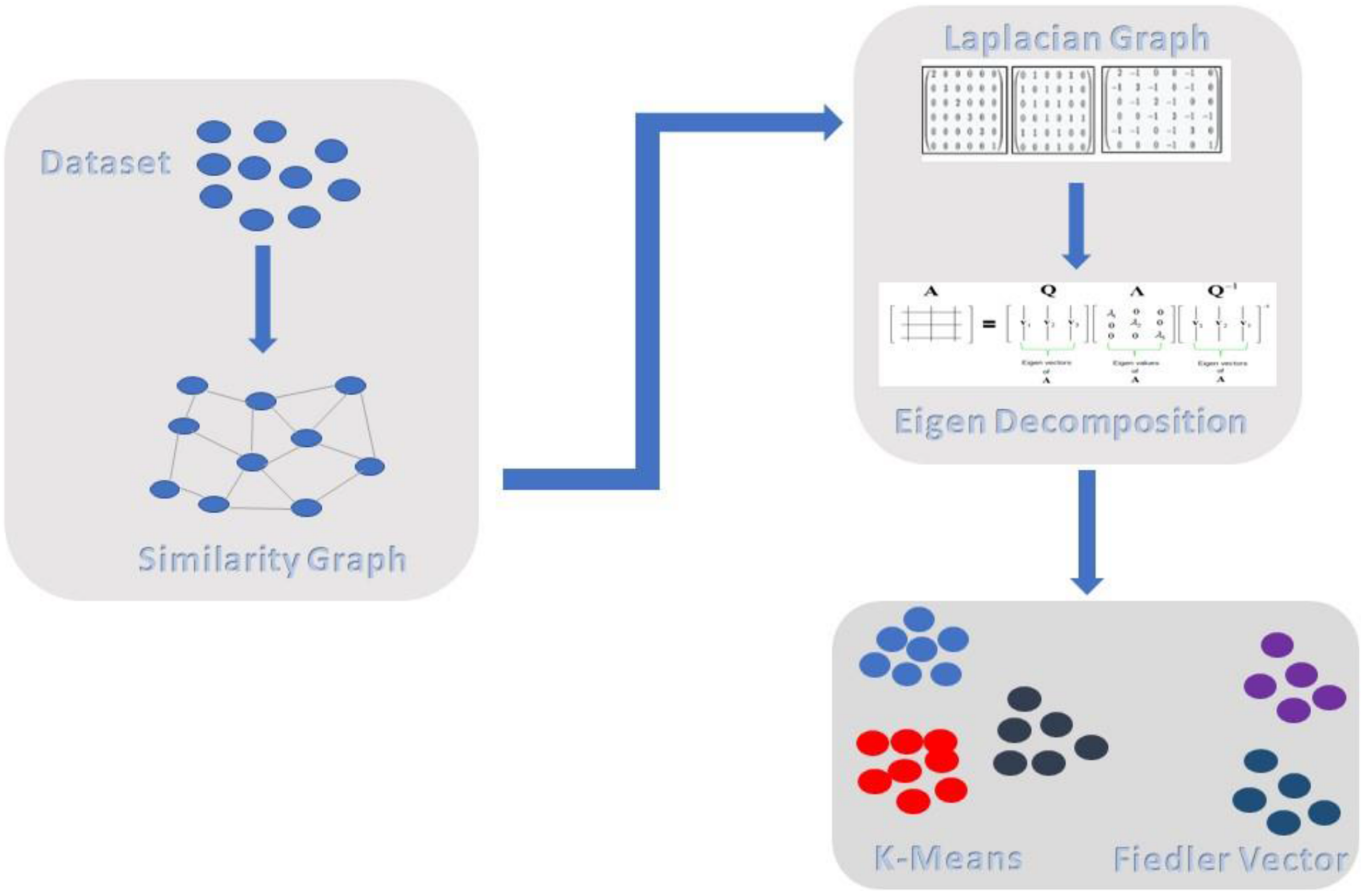
Proposed architecture.

The proposed method comprises of the following steps as shown in Fig. 2. In the first step, we do pre-processing, followed by building a similarity graph of the given dataset, which is the most important step in spectral clustering. Then, we introduce weighted K-NN for building the similarity graph. In the next step, we generate the graph Laplacian and for that we need to find the degree of the node and degree matrix. Decomposition is the next step in which we find the Eigen values and Eigen vectors of the Graph Laplacian. Finally, we cluster or group the data with the standard k-means technique. In Fig. 2, we can see the overall illustration of the proposed approach.

#### Pre-processing

The most significant and important phase in data mining is pre-processing the data. Distance-Based techniques such as K-NN, support vector machine, and k-means are probably the utmost techniques which are affected by the variety of features. For missing data, we implemented k-nearest neighbors which uses feature similarity. Values of any new data points can be predicted by using feature similarity. What we want to convey is that a new point is allotted a value based on the position of that point, how sharply it bears a resemblance to the points in the training data. It generates an elementary mean impute, and the resulting list is later used to formulate a KDTree. Next, it uses the KDTree to figure out nearest neighbours (NN). After finding the nearest neighbors, it holds the weighted average of the nearest neighbors. Consequently, we scaled our data before employing weighted-KNN in order to ensure that all attributes contribute equally to the outcome. To ensure this, we used standardization, in which it replaces the values by the z-scores by using the following Eq. (1).

(1) $${x}^{\prime} \equiv \displaystyle{{x - \bar x} \over x}$$

This restructures the features with standard deviation of 1 (*σ* = 1) and a mean of ‘0’ (= 0).

#### Similarity graph

In this step we built a similarity graph based on weighted K-NN, in the pattern of an adjacency matrix. An adjacency matrix is a form of square matrix which is to characterize a finite graph. The features of the matrix signify the adjacency of vertices in a graph. Suppose a graph (a simple one) the adjacency matrix is a square $\left| N \right|*\left| N \right|$ matrix S such that:

$${S_{mn}} = 1, \rm if\; m\; and\;n \; are\;connected\;(edge\;between\;m\;and\;n)$$

$${S_{mn}} = 0,\rm \;if\;m\;and\;n\;are\;not\;connected\;(no\;edge\;between\;m\;and\;n)$$

(2) $${S_{mn}} = \left\{ {\matrix{ {{w_{mn}} \to \rm weight\;of\;edge\;between\;m\;and\;n}  {} \cr {0 \to \rm if\;no\;edge\;in\;between\;m\;and\;n}  {} \cr } } \right.$$

In the matrix diagonal features are all zero because loops (edges from a vertex to itself) are not acceptable in minimal graphs. For the construction of Similarity Graph, we preferred to use an improved variant of k-nearest neighbors known as weighted k-NN proposed by Xu & WunschII (2005). Dudani in Pothen, Simon & Liou (1990) proposed the primary weighted approach for k-NN voting. In this method from the interval (0,1) weights are taken accordingly. The more the closer the more the data point will have weightage, the most nearby neighbor data point is weighted with 1, the outermost data point with 0 and as for the other data points, they are scaled among by the linear mapping illustrated in Eq. (3).

(3) $${w_i} = \left\{ {\matrix{ {\displaystyle{{{d_k} - {d_i}} \over {{d_k} - {d_i}}} \to {d_k} = {d_1}} \cr {1 \to {d_k} \ne {d_1}} \cr } } \right.$$

There are two additional alternatives suggested by Pothen, Simon & Liou (1990), the rank weight (Eq. (4)) and the inverse distance weight (Eq. (5)).

(4) $${w_i} = \displaystyle{1 \over {{d_i}}}$$

(5) $${w_i} = k - i + 1$$

Inverse of the squared distance can be more reliable instead of the inverse distance (Shi & Malik, 2000; Dudani, 1976; Mitchell, 1997). Probably, in both we may have a possibility of division by zero. We can solve it by addition of a negligible constant as shown in Eq. (4).

(6) $${w_i} = \displaystyle{1 \over {d_i^2 + \varepsilon }}$$

In Eq. (1) the exclusion of the Kth neighbor by the weighting function from the voting process in the condition when dk d1, since dk = 0 for i = k. Mitchell (1997) offers a simplification of the weighting function by presenting fresh parameters, *s* ≥ *k* and *a* ≥ 0. By using Macleod introduced parameters, we can conquer that deficiency. After the numerous combo previous parameters, which have been examined in Lucińska & Wierzchoń (2012), we will use s = k with a = 1.

(7) $${w_i} = \left\{ {\matrix{ {\displaystyle{{({d_s} - {d_i}) + a({d_s} - {d_1})} \over {(1 + a)({d_s} - {d_1})}} \to {d_s} = {d_1}} \cr {1 \to {d_s} \ne {d_1}} \cr } } \right.$$

#### Laplacian matrix

In this step we project the data onto a lower dimensional space. This measure deals with the possibility if some elements of the identical cluster may be not nearby in the provided dimension. Thus, the reduction of dimensional space takes place here so that the data points gets closer and thus those data points can be grouped together in the same cluster by a conventional clustering technique. This whole process is done through the procedure of computing the Laplacian Matrix. For the Laplacian Matrix computation we need to define degree of a node. The definition of the degree of *m*_*th*_ node is:

(8) $${d_m} = \sum\limits_{[n|(m,n)\varepsilon E]}^n {W_{mn}}$$

The Laplacian Matrix is defined as: L = D − A, where ‘D’ is the diagonal matrix of degrees and ‘A’ is the adjacency matrix defined by above equation. Symmetric normalized Laplacian matrix is described as following in Charikar (2002):

(9) $${L_{mn}} =\left\{ {\matrix { {1 \to \rm if\;m \;=\; n\;and\;{d_m} \ne 0} \cr { - \displaystyle{1 \over {\sqrt {{d_m}{d_j}} }} \to \rm if\;m\;and\;n\;are\;adjacent} \cr {0 \to \rm other} }}\right.$$

#### Decomposition

Spectral or eigen decomposition is the presentation in the form of factorization of a certain matrix into a recognised structure, therefore the matrix is characterized pertaining its eigen values and eigen vectors. Consequently, this factorization can be done only on diagonalizable matrices. A non-zero vector $\vec v$ of dimension N is an eigen vector of a square N × N matrix E, if it fulfils the equation:

(10) $$E\vec v = \lambda \vec v$$

where $\vec v$ is the eigen vector of matrix E corresponding to Lambda *λ*, and *λ* is scalar. Categorically, the eigenvectors are the set of vectors that E (linear transformation) purely lengthens or contracts, and the quantity that the linear transformation lengthens/contracts by is the eigenvalue. Equation (10) is known as the eigenvalue problem or equation. Computing the initial q eigenvectors of the Laplacian graph. The major eigenvalue of E correlate to the minimum eigenvalue of L, so we found our first eigenvector by this way. Moreover, for the next values, we recommend using the Von Mises iteration or Power Method because of its time complexity which is *O*(*n*^2^).

#### Grouping and clustering

In this section, finally, we implement an ordinary k-means algorithm on the newly achieved set of vectors of reduced dimension for the final clustering and grouping. K-means is a technique of clustering vector quantization, formerly from signal processing, that intent to panel ‘n’ observations into ‘k’ groups in which each observation belongs to the cluster with the nearest mean cluster centres or cluster centroid, serving as a prototype of the cluster. The goal of the clustering technique ‘k-means’ is to classify concealed variables of the large amount of data. The underlying or hidden variables are the centroids of clusters of that data-set. Through the smallest distance the representatives of a cluster or group are determined of each data to the centroid of the cluster.

## Results and Discussions

We made a comparison of our work with the original work (Wu et al., 2008) on two datasets. The original work uses sign-less Laplacian matrix and mutual k-nearest neighbors, whereas we use Symmetric normalized Laplacian matrix and weighted k-nearest neighbors. Our proposed algorithm uses the same properties of eigenvectors as of the original work. For the purpose of comparison, the utmost performance of our algorithm the parameter *σ* values were selected manually, as defined by Fischer & Poland (2004). We preferred to use their values. The algorithms are assessed on two datasets, they cap an extensive variety of complexities. One is artificial dataset and other is real world problem. Artificial dataset “blobs” is generated by diffusing the data points by means of Gaussian distribution.

**Figure 3:**
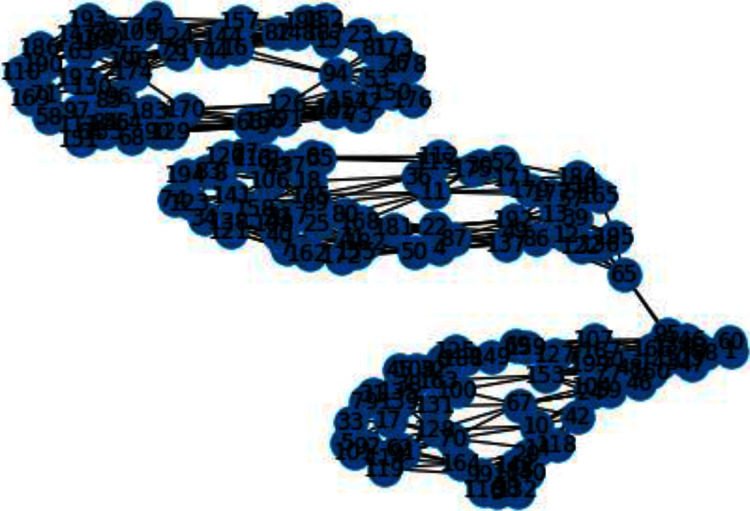
K-NN constructed similarity graph.

**Figure 4:**
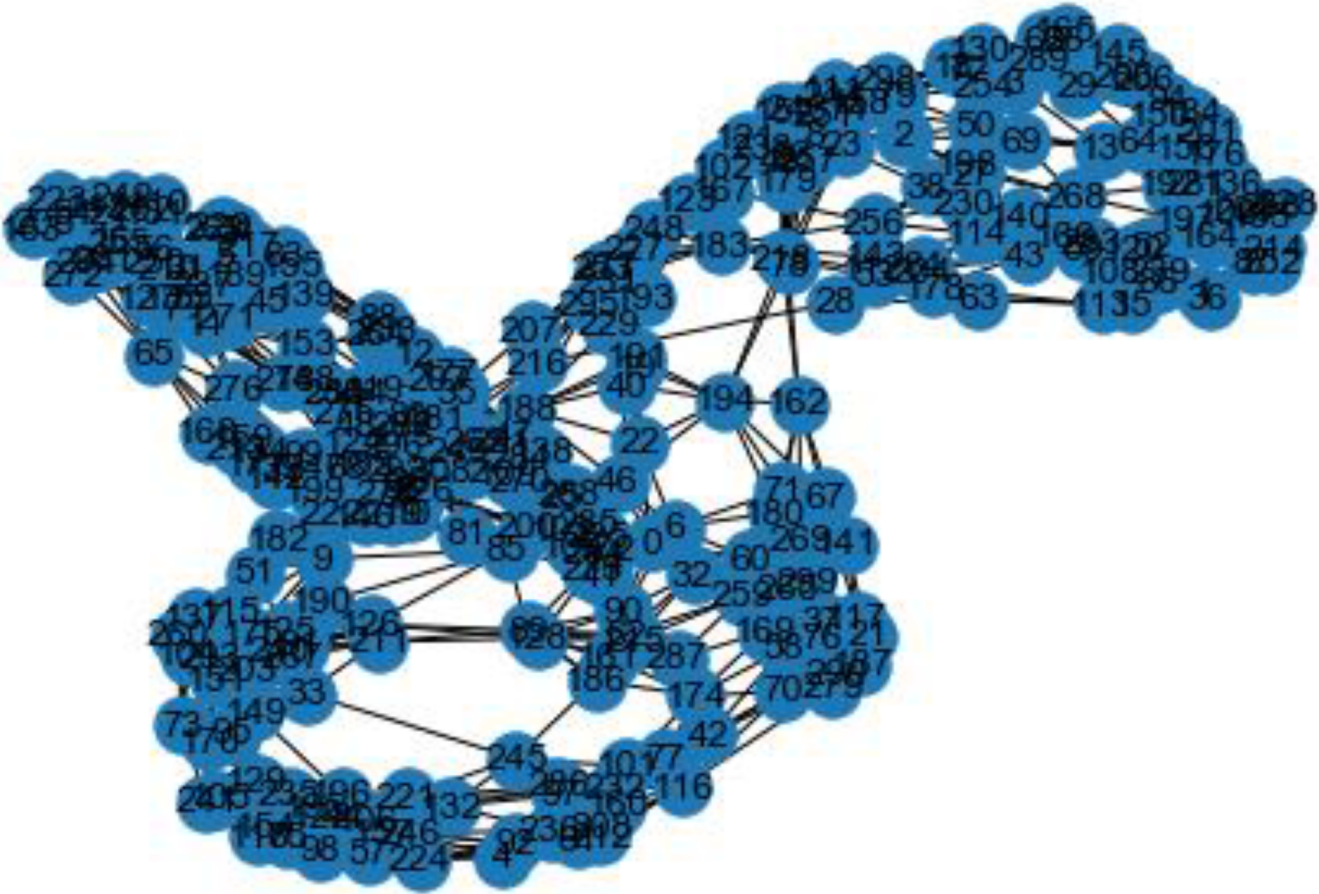
Weighted K-NN constructed similarity graph.

In Figs. 3 and 4, we can observe the difference of using different metrics for generating similarity graphs.

**Figure 5:**
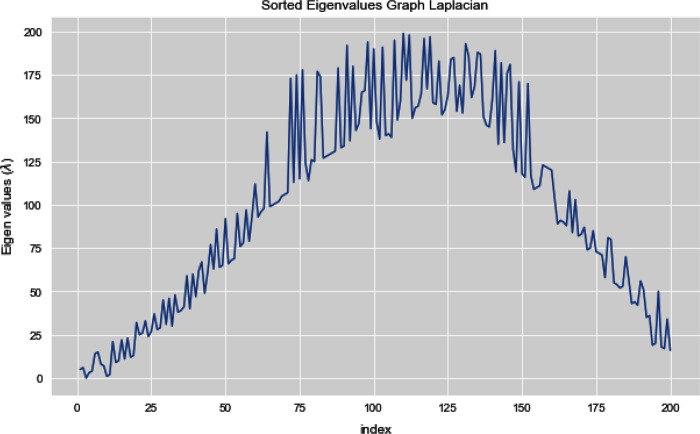
Sorted eigen values of graph Laplacian using K-NN.

**Figure 6:**
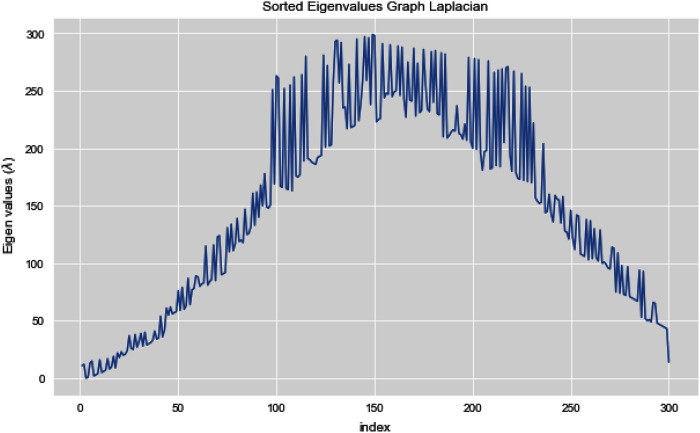
Sorted eigen values of graph Laplacian using weighted K-NN.

Figures 5 and 6 illustrates all the sorted eigen values of the given dataset. Figure 5 shows the sorted eigen values resulted through k-nearest neighbors graph and Fig. 6 illustrates the sorted eigen values resulted by our proposed technique weighted k-nn. We can see a clear difference between both the methods.

**Figure 7:**
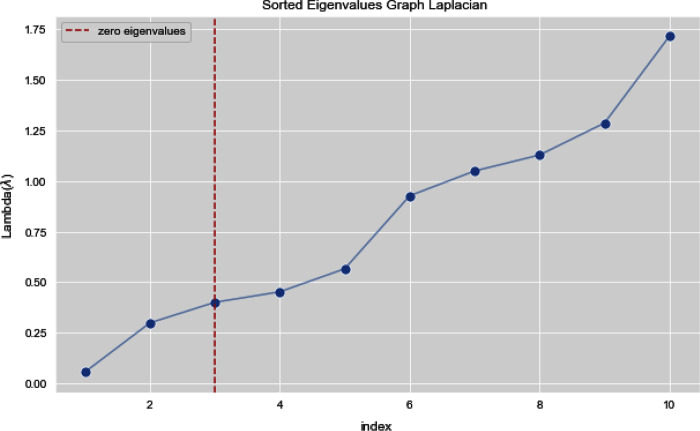
First 10 sorted eigen values of graph Laplacian using K-NN.

**Figure 8:**
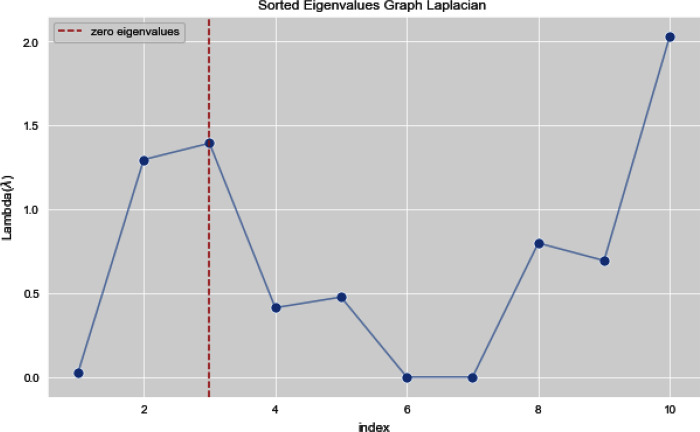
First 10 sorted eigen values using weighted K-NN.

In Figs. 7 and 8, we observe the first ten eigenvalues of both the algorithms and then for these eigenvalues we consider their respected corresponding eigen vectors. Overall, resulting zero or less than zero eigenvalues, is too restraining when the groups or clusters are not segregated allied (connected) components. In order to deal with this, we define the number of groups or clusters we need to discover. Occasionally it is essential to place eigenvalues in lowest to highest order. But then we also may need to rearrange the eigen vectors so they still go with the same eigenvalues. We sort the eigenvalues and keep the corresponding values of eigenvalues and their vectors.

**Figure 9:**
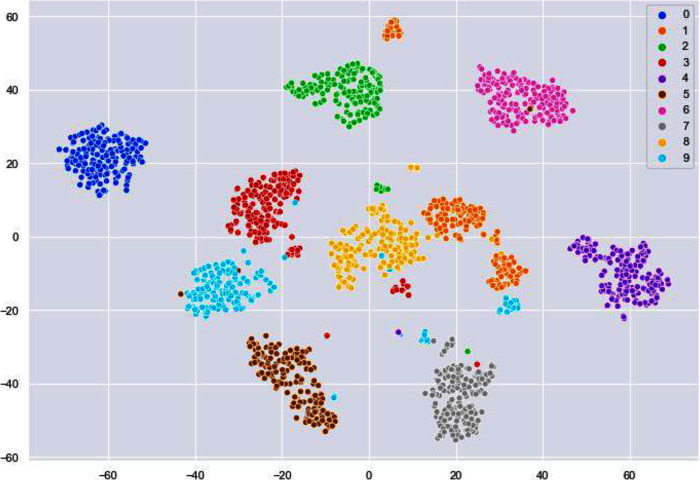
Clusters illustrating different digits.

We have used k-means algorithm for the final clustering as it can be seen in Fig. 9. The reason behind using k-means is to get more than two clusters that is in this case the numbers 0–9.

**Figure 10:**
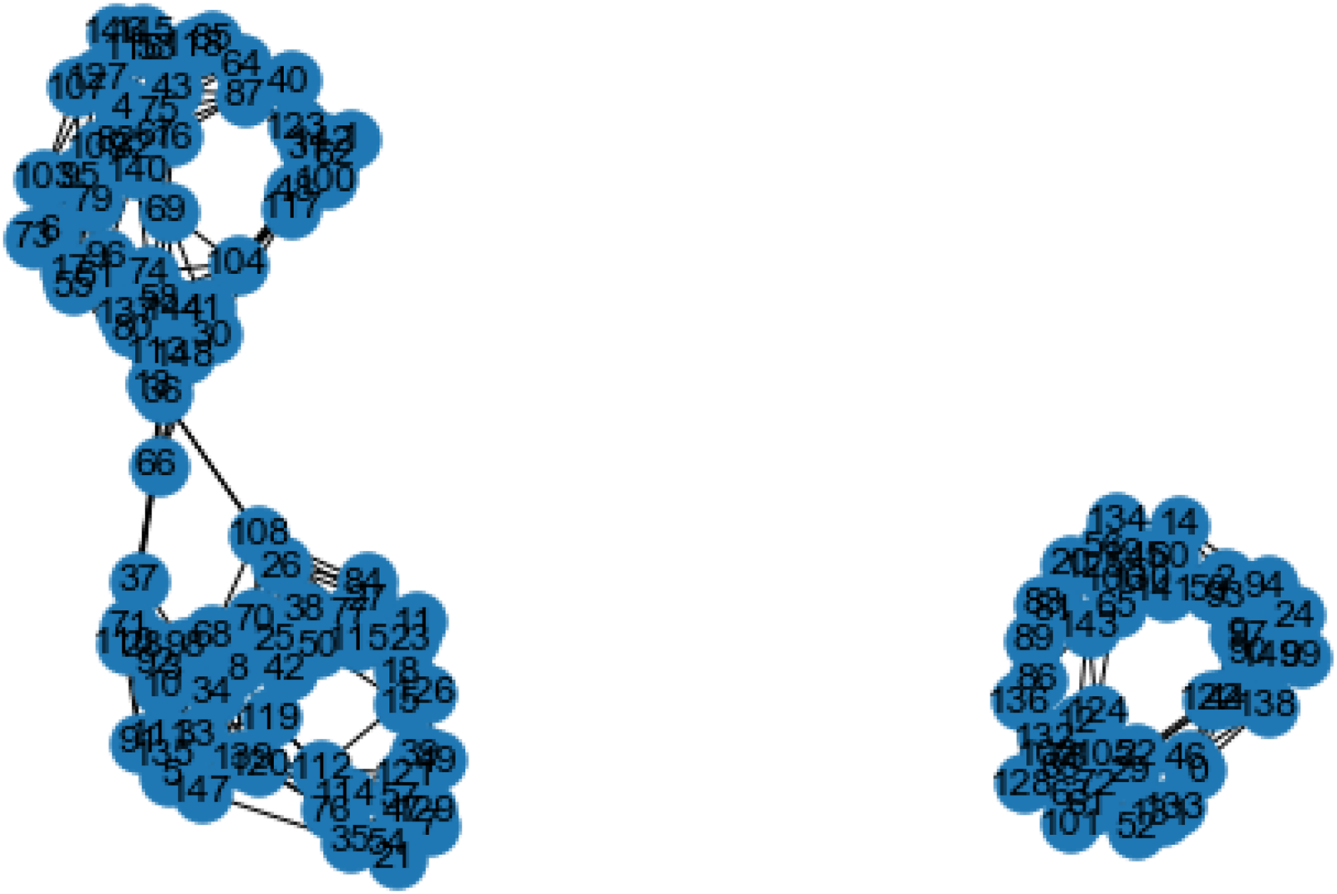
K-NN constructed similarity graph.

**Figure 11:**
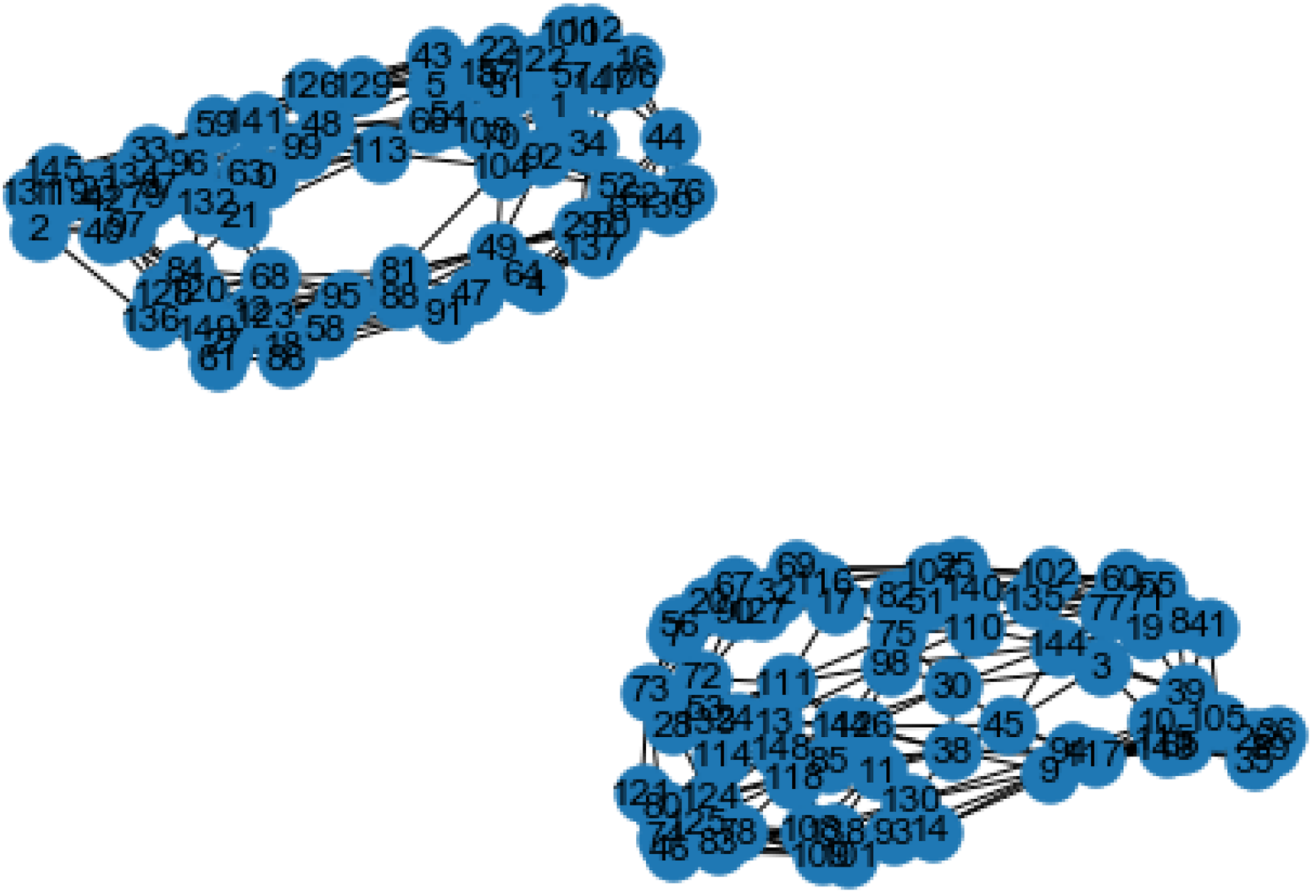
Weighted K-NN constructed similarity graph.

Now we will discuss the results generated from the artificial data set blobs. Artificial data set “blobs” is generated by diffusing the data points by means of Gaussian distribution. Figures 10 and 11 represents the similarity graphs produced by using k-nearest neighbors graph and the proposed affinity matrix weighted k-nearest neighbors graph.

The reason behind use of weighted k-nn for generating the similarity measure is one of the various problems that influence the achievement and performance of the K-nearest neighbors is the optimal value of the hyper parameter “k”. If we take k too small, the algorithm would be too susceptible to outliers. And, if “k” is very large, then there are chances that the neighborhood might comprise a lot of data points from different classes, as the problem can be seen in Figures 10 and 11. Also in Figs. 10 and 11, the difference between both the similarity graph construction is visible. Additionally, there may be an issue of combining the class labels in k- Nearest Neighbors approach. Taking the majority vote is the simplest method of all, but there is probability of a problem if the distance between nearest neighbors varies widely and the neighbouring data points more accurately point out the class of the object.

**Figure 12:**
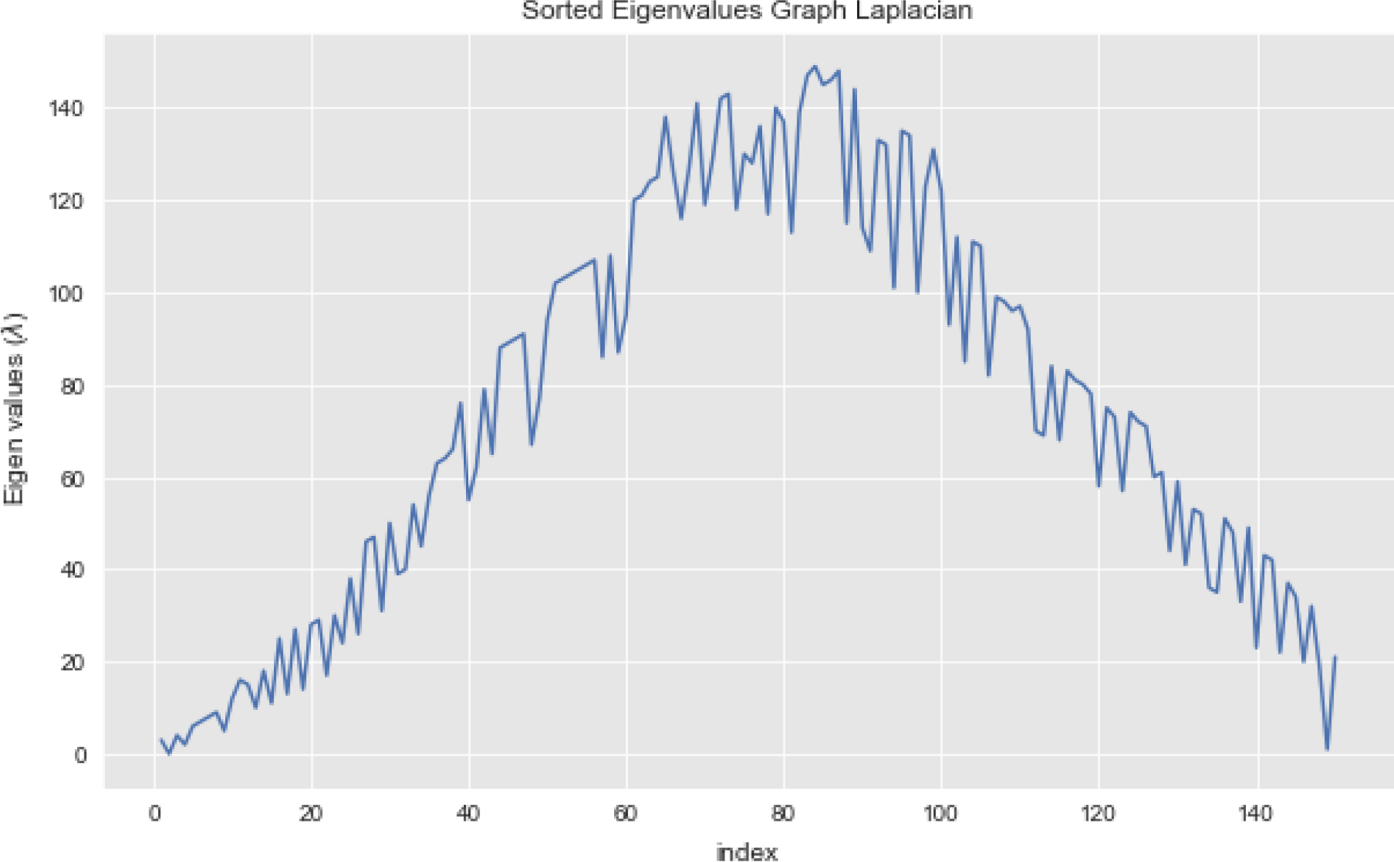
All sorted eigen values of dataset 2.

In Fig. 12, we can see all the sorted eigen values of Graph Laplacian. Using Graph Laplacian we want to make the structure of the data obvious so that we can make the clusters in the data clear and obvious.

**Figure 13:**
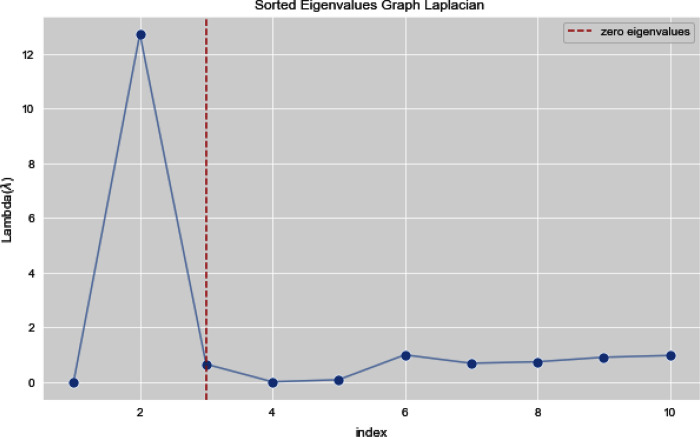
First 10 sorted eigen values of K-NN.

**Figure 14:**
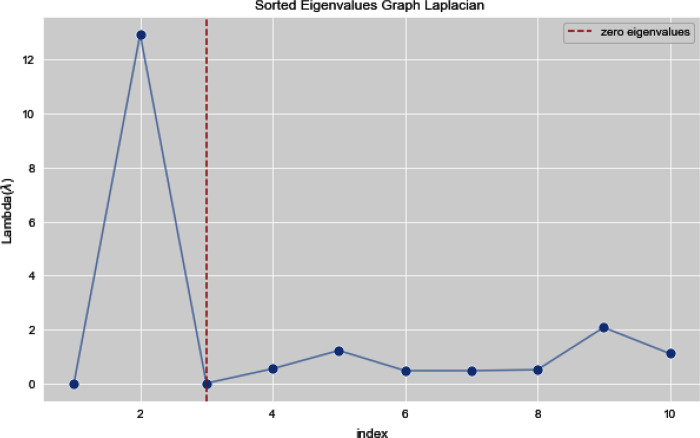
First 10 sorted eigen values of weighted K-NNFi.

Figures 13 and 14 illustrates the first 10 sorted eigen values of Graph Laplacian, we generate the eigen values with both the techniques k-nearest neighbors graph and weighted k-nearest neighbors graph.

**Figure 15:**
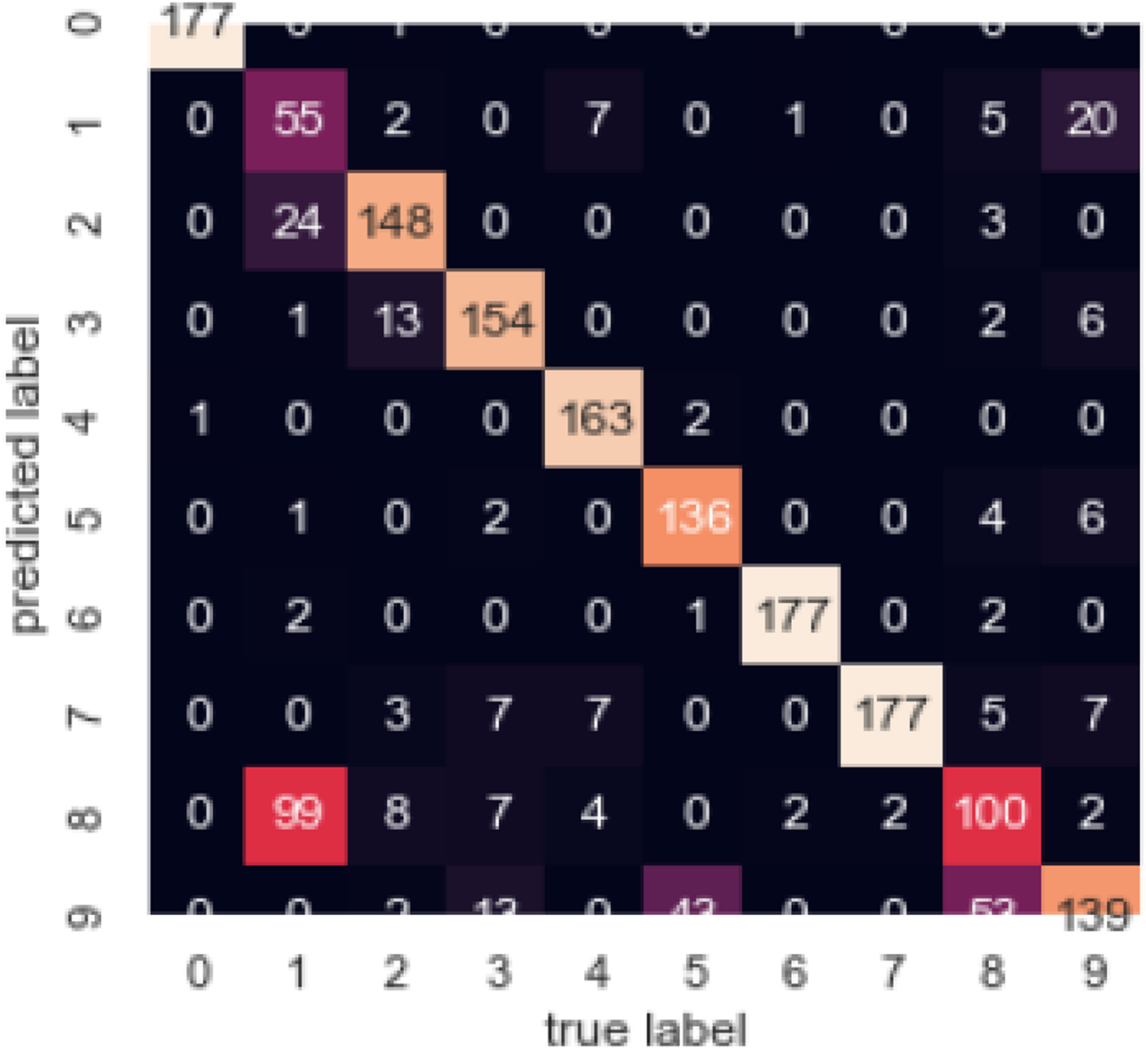
Confusion matrix.

Figure 15 illustrates the confusion matrix, by which we can observe the correlation between different attributes of the dataset.

**Figure 16:**
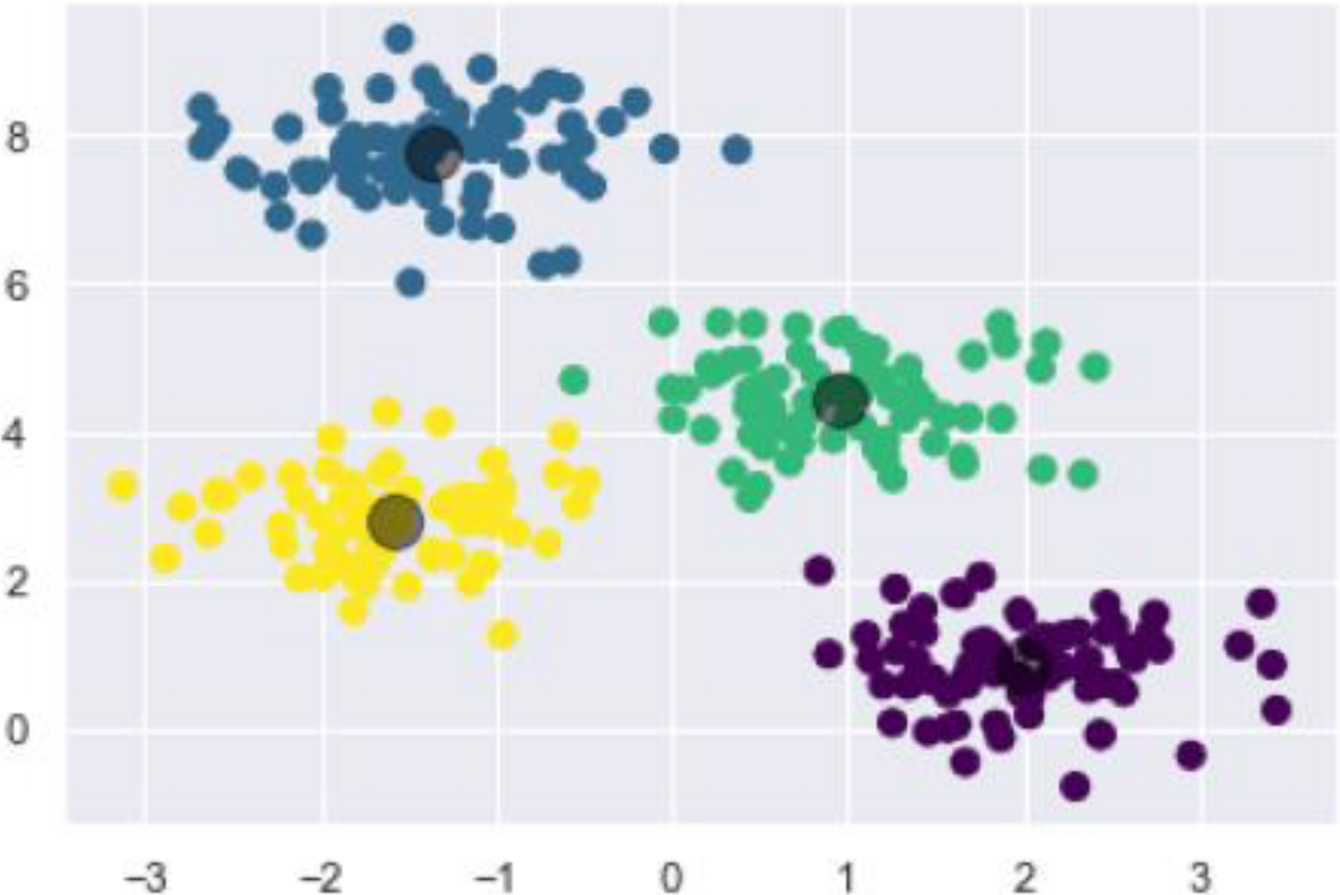
Clusters illustrating different digits.

In Fig. 16, for the final grouping or clustering we used the Fiedler vector to partition the data points. Eigen vector that corresponds to smallest eigen value (non-zero) is the Fiedler vector. Cluster one contains the indices values below zero and rest of the values are assigned to the second cluster. Association of indicator vector with each eigen value (non-zero) of a matrix is must. Indicator vector (individual one) perfectly comprises binary values to specify clusters association, as well as they are orthogonal to each other.

In Table 1, we compared different algorithms for spectral clustering. Our algorithm stands tall among all of these.

**Table 1:** Comparison of our algorithm with other algorithms.

Algorithm	Homogeneity	Completeness	V Measure
LSC-R	0.53	0.84	0.65
LSC-K	0.55	0.88	0.69
Speclus (K-NN based)	0.56	0.94	0.73
W-KNN based Spectral Clustering	0.58	0.98	0.78

## Conclusion

In this paper, we have introduced a new similarity metric known as weighted k-nearest neighbors for the construction of the affinity matrix. It is constructed based-on the weighted K-NN in which we give weight-age to every node based on how far or near the nodes are. If the node is near-by it is given greater weight-age and if it is far away it’s given less weight-age.

Our experimental results shows that a good similarity metric is very significant for spectral algorithm in order to achieve good results. Our results shows that our technique is good enough to cluster the data in a good way.

## Supplemental Information

10.7717/peerj-cs.692/supp-1 Supplemental Information 1 Affinity Matrix Code.

## Additional Information and Declarations

### Data Availability

The following information was supplied regarding data availability: The data is available in KAGGLE: https://www.kaggle.com/oddrationale/mnist-in-csv. The affinity matrix source code is available as a Supplemental File.
